# Integrated Multi-Omic Analysis Reveals Immunosuppressive Phenotype Associated with Poor Outcomes in High-Grade Serous Ovarian Cancer

**DOI:** 10.3390/cancers15143649

**Published:** 2023-07-17

**Authors:** Russell Keathley, Masha Kocherginsky, Ramana Davuluri, Daniela Matei

**Affiliations:** 1 Department of Obstetrics and Gynecology, Feinberg School of Medicine, Northwestern University, Chicago, IL 60611, USA; russell.keathley@northwestern.edu (R.K.); mkocherg@northwestern.edu (M.K.); 2 Driskill Graduate Program in Life Sciences, Feinberg School of Medicine, Northwestern University, Chicago, IL 60611, USA; 3 Department of Preventive Medicine (Biostatistics), Feinberg School of Medicine, Northwestern University, Chicago, IL 60611, USA; 4 Robert H. Lurie Comprehensive Cancer Center, Chicago, IL 60611, USA; 5 Department of Biomedical Informatics, School of Medicine, Stony Brook University, Stony Brook, NY 11794, USA; ramana.davuluri@stonybrookmedicine.edu; 6 Jesse Brown VA Medical Center, Chicago, IL 60612, USA

**Keywords:** high-grade serous ovarian cancer (HGSOC), multi-omic analysis, data integration, consensus clustering, neural network

## Abstract

**Simple Summary:**

Tumor classification based on genomic features may be able to identify new clinically relevant subtypes and disease characteristics. By integrating multiple levels of genetic and epigenetic data, distinct clusters can be defined among tumors with similar histology and previously unknown distinguishing features. Here, we aimed to find prognostic subtypes among high-grade serous ovarian tumors by integrating their transcriptomic and methylomic features. Feature selection was applied to retain only those features most significantly correlated with disease recurrence. By using consensus clustering and machine learning algorithms, we describe four groups of tumors characterized by unique genetic and epigenetic properties which were associated with significant differences in clinical outcomes. By using both techniques in succession, we uncovered both differential features between groups and defining ontologies therein. One such group was associated with stromal and immune diverse cell populations and was associated with poor clinical outcomes. Our findings identify unique contributors to disease recurrence in high-grade serous ovarian cancer.

**Abstract:**

High-grade serous ovarian cancer (HGSOC) is characterized by a complex genomic landscape, with both genetic and epigenetic diversity contributing to its pathogenesis, disease course, and response to treatment. To better understand the association between genomic features and response to treatment among 370 patients with newly diagnosed HGSOC, we utilized multi-omic data and semi-biased clustering of HGSOC specimens profiled by TCGA. A Cox regression model was deployed to select model input features based on the influence on disease recurrence. Among the features most significantly correlated with recurrence were the promotor-associated probes for the NFRKB and DPT genes and the TREML1 gene. Using 1467 transcriptomic and methylomic features as input to consensus clustering, we identified four distinct tumor clusters—three of which had noteworthy differences in treatment response and time to disease recurrence. Each cluster had unique divergence in differential analyses and distinctly enriched pathways therein. Differences in predicted stromal and immune cell-type composition were also observed, with an immune-suppressive phenotype specific to one cluster, which associated with short time to disease recurrence. Our model features were additionally used as a neural network input layer to validate the previously defined clusters with high prediction accuracy (91.3%). Overall, our approach highlights an integrated data utilization workflow from tumor-derived samples, which can be used to uncover novel drivers of clinical outcomes.

## 1. Introduction

Ovarian cancer remains among the leading causes of cancer death among women, with an overall 1 in 78 mortality risk [1]. High-grade serous ovarian cancer (HGSOC) is the most prevalent subtype, with a 5-year survival rate of approximately 47.4% [2]. HGSOC is thought to originate from the fallopian tube epithelium and harbors—almost universally—a p53 mutated signature. These tumors exhibit heterogeneous gene expression and methylation profiles and a diverse mutational landscape [3]. The first-line treatment is a combination of platinum-based chemotherapy, with ~85% of patients achieving a complete or partial response to therapy [4]. However, recurrence rates are high, with a median platinum-free interval (PFI) of 18 months [3]. Patients whose recurrence occurs earlier than 6 months are deemed “platinum-resistant”, and those recurring after are “platinum-sensitive”. Although other second-line therapies, such as liposomal doxorubicin, bevacizumab, and gemcitabine, have demonstrated clinical benefit, others—such as PD1/PD-L1-targeted immunotherapies—have yielded only marginal responses [5,6,7,8].

The integrated molecular analysis of HGSOC in The Cancer Genome Atlas (TCGA) yielded a classification of tumors based on histology and genomic features (mutation, copy number, gene expression) [9]. This analysis found ubiquitous *TP53* mutations, a defined subset of tumors displaying *BRCA1/2* germline mutations, and characteristic DNA copy number alterations and promoter methylation events. Non-negative matrix factorization (NMF) consensus clustering was conducted using mRNA expression, miRNA expression, DNA copy number, and promoter methylation data as input. Four subtypes were then defined: Mesenchymal, Proliferative, Immunoreactive, and Differentiated, building off a prior analysis which used microarray in isolation [9,10]. The Mesenchymal subtype demonstrated poor overall survival, increased expression of genes related to stromal components, and active EMT- and angiogenesis-related genes. The Proliferative subtype showed increased expression of markers of proliferation (e.g., *MCM2*, *PCNA*), as well as increased *WNT*/β-catenin signaling [9,10]. Tumors with an Immunoreactive subtype were associated with improved overall survival and upregulated genes relating to immune cell activation (*CXCL11*, *CXCL12 CXCR3*, etc.). The Differentiated subtype displayed high expression of markers of epithelial differentiations, such as *MUC1* and *MUC16.* Other studies have attempted similar “omic” classifications; however, the prognostic and therapeutic relevance of molecular subtypes remains variable between studies and has not drastically influenced treatment decisions [9,10,11,12].

Advancements in unsupervised learning and multi-omic data integration are allowing for more complex methods of tumor subtyping [13,14]. These newer subtyping methods can capture more disease context with each added layer of data but remain untested for clinical utility. Here, we present a novel workflow using integrated methylomic and transcriptomic data from TCGA, which we use to cluster HGSOC tumors by using “omic” features selected based on correlation with disease recurrence. We characterize the genomic features of these newly defined clusters through downstream differential analyses and use machine learning techniques to uncover novel subtypes with distinct phenotypes and clinically relevant signatures.

## 2. Materials and Methods

### 2.1. Data Collection

Paired RNA-seq and 27k Illumina methylation array data were obtained for 374 HGSOC specimens from TCGA, downloaded directly from the Genomic Data Portal (GDC) via a 2011 Legacy dataset available online [15]. Legacy data were required, as cancer recurrence information is not available for current iterations. Supplemental clinical metadata was gathered using the Broad Institute GDAC Firehose database (http://gdac.broadinstitute.org), accessed on 17 August 2022. Time to recurrence was defined as days from diagnosis to tumor recurrence. Patients were censored at the last date they were alive and known to be recurrence-free.

### 2.2. RNA-seq Data Processing

RNA-seq data were collected as *BAM* genomic alignment files. Fragments per kilobase of transcript per million mapped reads (FPKM) and expected gene counts were then calculated using RNA-Seq by Expectation–Maximization (RSEM) [16]. FPKM counts were normalized to log2(FPKM + 1) and used for clustering analysis and neural network construction. Data distribution was examined after normalization to check for normality. Low gene counts were filtered to remove those with counts of zero in ≥70% of samples. RSEM expected counts were rounded to the nearest integer and used for downstream differential analysis. Three patients had multiple aliquot samples; in these cases, the mean expression values across aliquots were used.

### 2.3. 27k Methylation Array Data Processing

27k Illumina methylation array data were collected in a SeSAMe Methylation Beta Value file format. Nine multi-aliquot samples existed, for which the mean beta values were used to represent each sample. Missing beta values were imputed using ChAMP version 2.30.0, using the K-nearest neighbors method (KNN) with k = 5 neighbors [17]. Probes with a proportion of samples with an NA value above 0.2 were removed. For downstream correlation analyses, probes were averaged to the gene level.

### 2.4. Data Integration and Feature Selection

The expression data (count matrix) and methylation data (beta values matrix) were unified such that each patient had one representative sample in each dataset. Prior to sample clustering, various feature selection methods were performed to select the most relevant features for cluster distinction. Two main methods were used—mean absolute deviation (MAD) and Cox proportional hazards regression models [18].

The univariate Cox model was used for each feature (gene, probe) to indiscriminately test its significance in relation to disease recurrence. In this case, the event variable was the binary recurrence or censoring indicator, and the time was the number of days from cancer diagnosis to recurrence, death, or censoring. The Cox-selected features were chosen for downstream analysis. Features were evaluated for selection based on both R-square value and Ward *p*-value. A cutoff value of 0.05 was used for the proportional hazards *p*-value for each feature. The coxph function in R survival package was used for this analysis [19].

The MAD method was initially used for feature selection for the 500 most variable features from each dataset but was not deployed in downstream clustering. The following formula was used:

MAD = Σ|xᵢ − μ|/n, where

n is the number of samples, and

|xᵢ − μ| is the absolute deviation of feature xᵢ from the mean.

### 2.5. Unsupervised Clustering Analysis

In total, 1467 features (methylation = 650 CpG probes, expression = 817 genes) were selected for use in the clustering analysis. Various methods of unsupervised clustering were used to identify distinct subtypes using this feature set. The methods used for clustering included Ward hierarchical clustering, partition around medoids (PAM), Clara, and similarity network fusion (SNF). Ultimately, a combined SNF and consensus clustering (CC) was applied [20]. First, SNF was conducted to obtain the fusion matrix of sample similarity for the combined methylation and expression feature set. Next, the matrix was used as the input sample distance for consensus clustering, with k = 4 nearest neighbors. In total, 20 iterations were performed, and an alpha variance for the local model was set to 0.5. This iterative clustering was performed using the CancerSubtypes package [21].

### 2.6. Survival and Recurrence Analysis

Overall survival and time to recurrence were estimated using the method of Kaplan–Meier and compared between the identified clusters using the logrank test. With a sample size *n* = 370 and expecting to identify 3 or 4 clusters, we expected the cluster size to be approximately *n* = 100. Assuming approximately 80% event rate (i.e., approximately 160 events in the two clusters), a Cox proportional hazards model comparing time-to-event outcomes between two such clusters would provide 80% power to detect a hazard ratio HR = 1.56 when testing at two-sided α = 0.05. Adjusting for multiple comparisons and testing at α = 0.01, there would be 80% power to detect HR = 1.72.

### 2.7. Differential Expression and Methylation

To test for differences in expression and methylation among the responsive and non-responsive clusters, differential expression was performed using DESeq2 [22]. DESeq utilizes an empirical Bayesian method to assess differential expression for each gene, making specific cluster-wise comparisons (e.g., Cluster 4 vs. Cluster 1). An FDR-adjusted *p*-value of <0.05 (Benjamini–Hochberg correction) and absolute LFC (|>0.5|) were used as thresholds for significance. To test for methylation differences among the two clusters of interest, differential methylation was performed using limma [23]. Limma employs a linear model with an empirical Bayes procedure to make pairwise comparisons between one cluster and all others. An FDR-adjusted *p*-value of <0.05 (BH) was used as a cutoff for significance with an absolute delta *beta* value (|>0.10|). Gene annotation for each differential probe was obtained from the Illumina Human Methylation 27k Annotation. Enrichment analysis using differentially expressed and methylated genes was conducted using Enrichr [24].

### 2.8. MLP Neural Network Architecture

The selected features (*n* = 1467) used for unsupervised clustering were used as the input nodes for a multilayer perceptron (MLP) artificial neural network. The data were split into 80/20 training and testing sets (by % of samples), respectively. The sample labels were defined as the cluster numbers (i.e., subtypes) previously identified. The model architecture was as follows: one input layer (nodes = 1467), three hidden layers (nodes = 1000, 1000, 100), and one output layer (labels = 4). A layer dropout rate of 0.1 was used in the first layer. For the first four layers, the *relu* activation function was used. For the output layer, the *softmax* activation function was used. The *adam* optimizer function was used, with a learning rate of 0.0001. The *categorical crossentropy* loss function was used. Model accuracy was calculated based on the confusion matrix of predicted and actual class labels. Machine learning analysis was performed with Keras [25].

### 2.9. Gene Set Enrichment Analysis

The top features were extracted based on high neuron fire frequency within the assembled neural network model. Genes of interest were isolated from the trained model, based on those with highest feature weights and bias in aggregate. Gene ontology enrichment analysis was then performed using clusterProfiler (software package) version 4.8.1 [26].

### 2.10. Additional Downstream Analyses

Three additional downstream analyses were performed on the identified clusters to evaluate further signature differences among them. Correlation analysis was performed for each cluster separately to evaluate the correlation of gene-level methylation with expression, using MethylMix 2.0 [27]. Immune cell-type composition analysis was applied using Immunedeconv, (clusterProfiler version 4.8.1) on TPM counts, to assess these differences among each cluster [28].

### 2.11. Workflow Validation and Analysis

To validate the described workflow and demonstrate its utility on an external dataset, RNA-seq expression and 450k methylation array data were collected from the HGSOC dataset maintained by the International Cancer Genome Consortium (ICGC) [29]. Following requisite filtering and quality control, 61 tumor samples for which both transcriptomic and methylation data were available were selected for analysis. Expression filtering and normalization were performed as described, using normalized HTSeq counts. A more stringent *p*-value cutoff was applied to Cox feature selection (*p* < 0.01) to account for the higher number of probes included in the 450k array. All downstream analyses were conducted in the same fashion as the described workflow. To compare methylation probes from 450k array ICGC data to 27k array TCGA data, 450 k probes were limited to only those falling within +/− 1500 base pairs of TSS, representing the promoter region. However, all 450k probes were considered for feature selection.

## 3. Results

### 3.1. Unsupervised Clustering Analyses of Integrated DNA Methylation and RNA Sequencing Data

**Figure 1:**
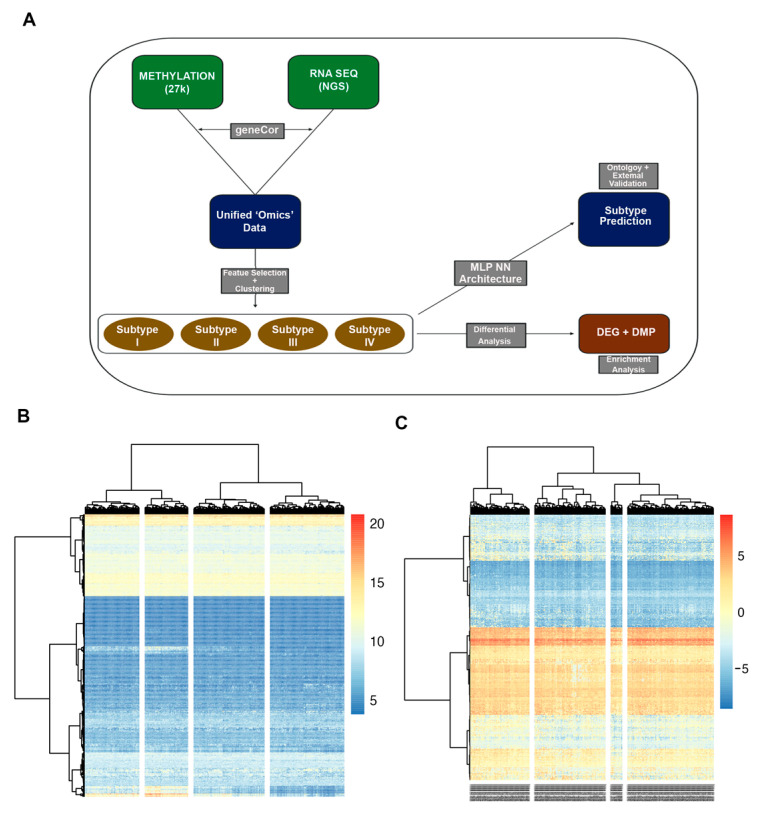
Analysis workflow (**A**). Methylation array (27k) and RNA sequencing data (RNA seq) datasets were unified to *n* = 370 patients. Necessary pre-processing, filtration, and normalization were performed for each dataset. (**B**,**C**) Ward clustering of unified RNA-seq (**B**) and methylation (**C**) data, for *n* = 370 patient samples. Feature selection using Cox regression resulted in 1467 features selected for clustering. K = 4 clusters set for analysis. Each column represents a tumor specimen, and each row represents a genomic feature. No major distinctive clusters observed using this method.

Data were processed and analyzed according to the workflow outlined in Figure 1A. Of the 380 patients within TCGA-OV dataset, for whom both methylation array and RNA-seq data were available, 370 samples for each assay remained after the unification and averaging of aliquots. Following filtering and normalization, a univariate Cox model (proportional hazards regression) was applied to evaluate the significance of each feature (gene and probe) related to disease recurrence. In total, 54,494 features were evaluated. The feature selection for the clustering analysis employed only those features with likelihood-ratio test *p*-values of <0.05. Prior feature selection attempts, using features with high variability (mean absolute deviation), were inconclusive.

In total, 1467 features were selected through the described algorithm and used for clustering. Among the features most significantly correlated with recurrence were the promotor-associated probes for the *NFRKB* and *DPT* genes and the *TREML1* gene (Supplementary Table S1). Ward hierarchical clustering was performed on the unified dataset, with k = 4 clusters (Figure 1B,C). No clear pattern of clusters emerged, possibly due to the high inter-sample variability of features. Thus, more sophisticated clustering methods were employed.

**Figure 2:**
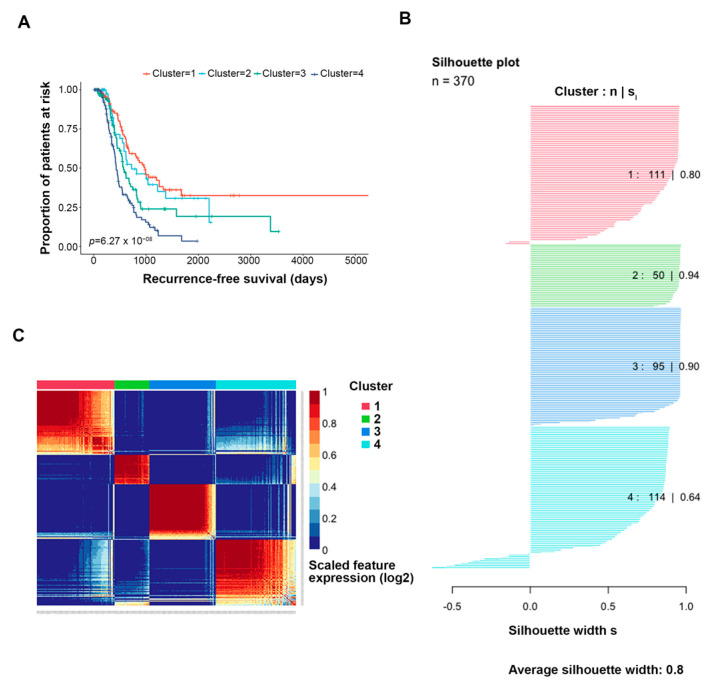
Clustering results, overview. Similarity network fusion plus consensus clustering (SNF + CC) was performed on the features selected by Cox regression (k = 817 genes, 650 probes). (**A**) KM-based time to recurrence analysis performed for each identified cluster. K = 4 clusters were optimal for significance (*p*-value 3 × 10^−7^). (**B**) The silhouette width of each identified cluster. (**C**) Heatmap of the called-feature expression of the features selected by Cox regression. Each column represents a patient, and each row represents a genomic feature.

Similarity network fusion combined with consensus clustering yielded optimal k = 4 clusters with distinct differences in disease recurrence propensity (Figure 2A). All identified clusters demonstrated a silhouette width of acceptable quality (Figure 2B). Called-feature expression was largely distinct by cluster (Figure 2C). Cluster 1 (*n* = 101 tumors) showed a higher propensity toward treatment sensitivity; the median time to disease recurrence was 977 days (Table 1). Cluster 4 (*n* = 111 tumors) showed a trend toward treatment resistance; the median time to disease recurrence was 428 days. Cluster 3 (*n* = 98 tumors) also trended toward resistance and lethality, with a median time to recurrence of 568 days and median time to death of 1248 days. These clusters were selected for downstream differential analyses, as they had the highest disparity in treatment outcomes.

### 3.2. Characteristics of Identified Clusters

Cluster 1: “Responsive”

**Figure 3:**
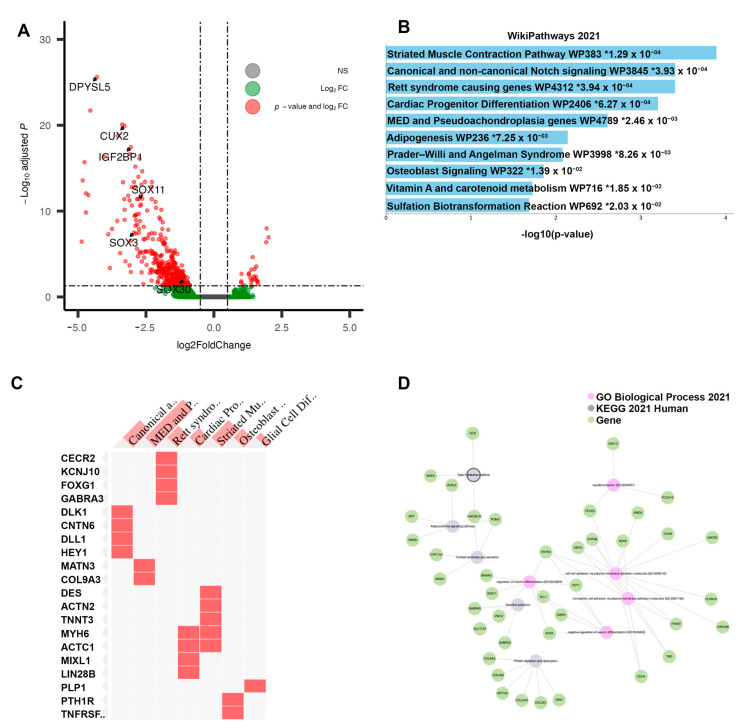
Results of differential analysis, Cluster 1. (**A**) All differentially expressed genes, with adj. *p*-value < 0.05 and |LFC| > 0.50, with genes of interest highlighted. (**B**) Pathway enrichment analysis for Cluster 1 (“Responsive”), performed using ENRICHR. Enriched pathways found using differentially expressed genes within Cluster 1. * denotes statistical significant (*p* < 0.05) (**C**) Presence of differentially expressed genes within significantly enriched pathway sets. (**D**) Network analysis using three foundational pathway sets, showing connectivity between differentially expressed genes and enriched pathways.

Differential gene expression for this subtype was performed against all others to identify features potentially contributing to chemo-responsiveness. In total, 407 genes (DEGs) were found to be differentially expressed (FDR-adj *p*-value < 0.05, |log2FC| > 0.50), 26 upregulated and 381 downregulated genes (Figure 3A). Of these, 261 were protein coding genes. All top 20 differentially expressed genes (by adjusted *p*-value) were downregulated in this cluster vs. all others (Table 2). The pathway analysis of the differentially expressed genes identified several enriched signaling pathways: Notch signaling, Adipogenesis, Sulfation Biotransformation, and Vitamin A and carotenoid metabolism (Figure 3B). Several of the differential genes found in the Notch signaling pathway have endogenous roles in development but also in differentiation and proliferation—*DLK1*, *CNTN6*, *DLL1*, and *HEY1* (Figure 3C). As observed in the network analysis, several sub-groups of differentially expressed genes form small clusters of network-oriented signaling (Figure 3D). Methylation analysis comparing Cluster 1 to all others revealed 1292 differentially methylated probes (DMPs), all limited to the promoter region, as included in the 27k Illumina platform. The top 20 DMPs, selected based on adjusted *p*-value, were all hypermethylated. In total, 41 genes were both differentially expressed and had at least one promoter-level probe differentially methylated. These genes were selected as a cluster “signature” for further inspection.

Cluster 3: “Non-Responsive|Immunosuppressive”

**Figure 4:**
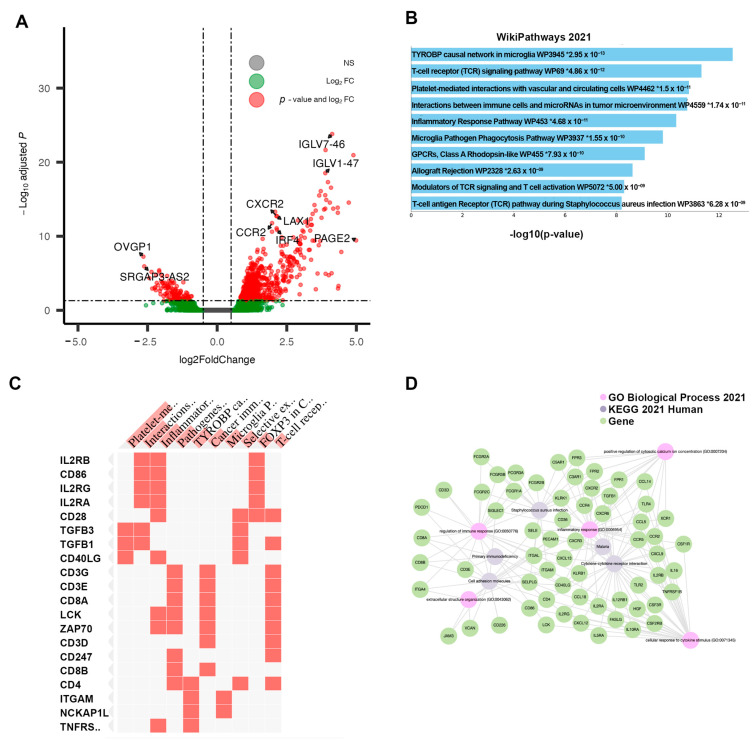
Results of differential analysis, Cluster 3 (“Non-Responsive|Immunosuppressive”) vs. Cluster 1 (“Responsive”). (**A**) All differentially expressed genes, with adj. *p*-value < 0.05 and |LFC| > 0.50, with genes of interest highlighted. (**B**) Pathway enrichment analysis for Cluster 3, performed using ENRICHR. Enriched pathways found using differentially expressed genes within Cluster 3. * denotes statistical significant (*p* < 0.05) (**C**) Presence of differentially expressed genes within significantly enriched pathway sets. (**D**) Network analysis using three foundational pathway sets.

**Figure 5:**
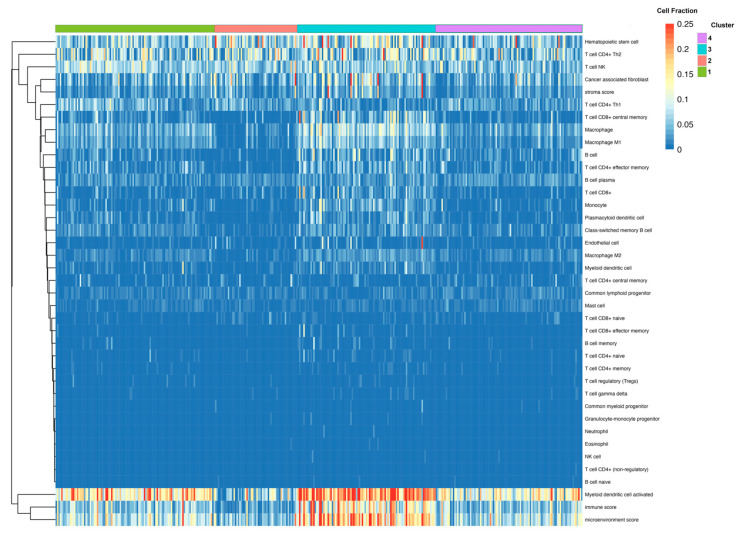
Fractional estimates of cell-type composition within each identified cluster. Constructed using immunedeconv R package. Higher fraction of multiple immune cell types found within Cluster 3.

The differential expression of Cluster 3, compared directly to the Responsive Cluster 1, revealed 826 DEGs—711 were upregulated and 115 were downregulated. Of these, 551 were protein-coding genes. Within the top 20 DEGs, sorted by adjusted *p*-value, many immunoglobulin heavy-chain genes (IgHV) and immunoglobulin kappa variable chains (IgKV) were identified (Table 3). Among the top protein-coding DEGs, several chemokine receptors were identified (e.g., *CXCR2*, *CCR2*) (Figure 4A). The pathway enrichment of these DEGs yielded multiple pathways related to immune and inflammatory signaling: Inflammatory Response Pathway, Chemokine signaling pathway, and Selective expression of chemokine receptors during T-cell polarization (Figure 4B). Groups of interleukin and costimulatory-coding genes defined the phenotype, preset across multiple enriched pathways (Figure 4C). No clear sub-clustering of differential genes was observed in the network analysis (Figure 4D). Due to the observed changes in immune/inflammatory signaling within this cluster, cell-type composition analysis was performed using Immunedeconv. The analysis estimated that Cluster 3 was enriched in immune cells, including both immune reactive cells like CD4+ effector memory T-cells and CD8+ central memory T-cells as well as immuno-suppressive cells, such as macrophages, M1 macrophages, M2 macrophages, monocytes, myeloid dendritic cells, cancer-associated fibroblasts, and hematopoietic stem cells (Figure 5). The overall myeloid dendritic cell activation score, the tumor microenvironment score, and the immune score were increased in Cluster 3 compared with Clusters 1, 2, and 4, suggesting that this cluster is associated with an immune-diverse phenotype. A Kruskal–Wallis test revealed statistically significant immune cell-type enrichment, particularly in Cluster 3 (Supplementary Table S2). Differential methylation analysis of Cluster 3 revealed 596 DMPs with an adjusted *p*-value < 0.05 and an absolute delta beta value ≥0.10. Of these, 354 CpG sites were hypermethylated, and five probes had an absolute delta beta value >0.19.

Cluster 4: “Non-Responsive|Hypomethylated”

**Figure 6:**
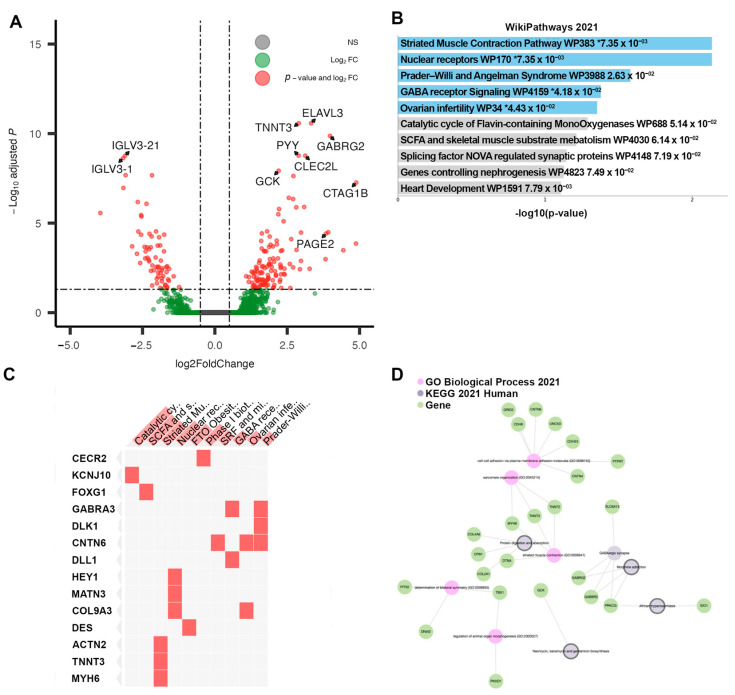
Results of differential analysis, Cluster 4 (“Non-Responsive|Hypomethylated”) vs. Cluster 1 (“Responsive”). (**A**) All differentially expressed genes, with adj. *p*-value < 0.05 and |LFC| > 0.50, with genes of interest highlighted. (**B**) Pathway enrichment analysis for Cluster 4, performed using ENRICHR. Enriched pathways found using differentially expressed genes within Cluster 4. * denotes statistical significant (*p* < 0.05) (**C**) Presence of differentially expressed genes within significantly enriched pathway sets. (**D**) Network analysis using three foundational pathway sets.

Cluster 4, associated with the shortest time to recurrence, contained tumors displaying 225 DEGs when compared directly to the Responsive Cluster 1. Of those, 161 genes were upregulated. In contrast to Cluster 3, IgHV and IGLV genes found among the DEGs of Cluster 4 were mostly downregulated (Table 4). However, several noteworthy genes were significantly upregulated (Figure 6A). The pathway enrichment of Cluster 4 DEGs revealed multiple pathways of interest unique to the cluster: GABA receptor signaling (*GABRA3* and *DLL1* genes), Ovarian infertility (*CNTN6* and *COL9A3*), and Nuclear receptors *(HEY1, MATN3, COL9A3)*, pointing to a phenotype more dysregulated in hormone signaling (Figure 6B–D). Differential methylation analysis of this cluster relative to Cluster 1 revealed 700 DMPs, all of them hypomethylated. Thus, we refer to this cluster as the “hypomethylated” cluster, including tumors with potentially less gene silencing occurring at DMP loci. The pathway analysis of the DMPs in this cluster revealed the enrichment of PI3K-Akt-MTOR signaling.

### 3.3. Workflow Validation on External Dataset

To validate the feature selection and clustering approach, we applied our methodology to an external ICGC clinical dataset, where time to disease recurrence was available [29]. Following Cox-based feature selection, 6884 gene features from RNA sequencing and methylation array data were used for SNF and consensus clustering (statistically significant correlation with disease recurrence, *p* < 0.01). Of these, 6505 features were methylation probes. A four-cluster optimal solution was again identified (Supplementary Figure S1). Each identified cluster had a significant difference in time to disease recurrence among clusters (*p* < 0.0001). In total, 56 promoter-region probes and 9 gene features overlapped with those identified in the primary model (Supplementary Table S3).

### 3.4. Pre-Biased MLP Neural Network

**Figure 7:**
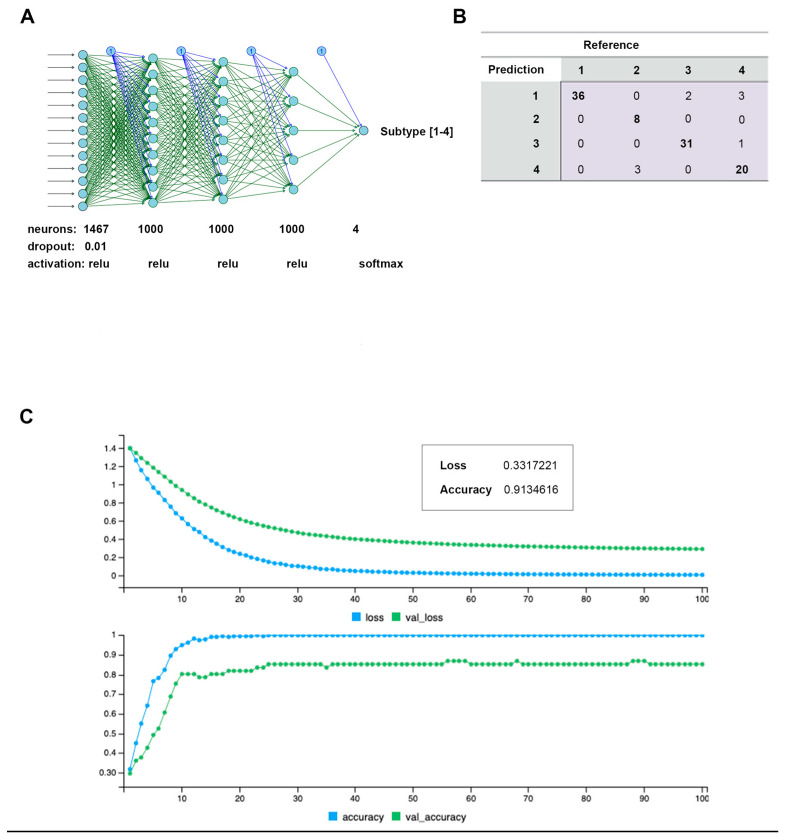
Multi-Layer Perceptron (MLP) neural network constructed to predict de novo clusters using features selected by Cox regression. (**A**) Architecture and tuning parameters used for final model construction. Four nodes in final (prediction) layer representing each cluster. A total of 1467 input features from Cox regression selection. (**B**) Confusion matrix generated by 80/20 model training/testing prediction of cluster ID. (**C**) Model training over 100 epoch period, with generated loss and prediction accuracy.

**Figure 8:**
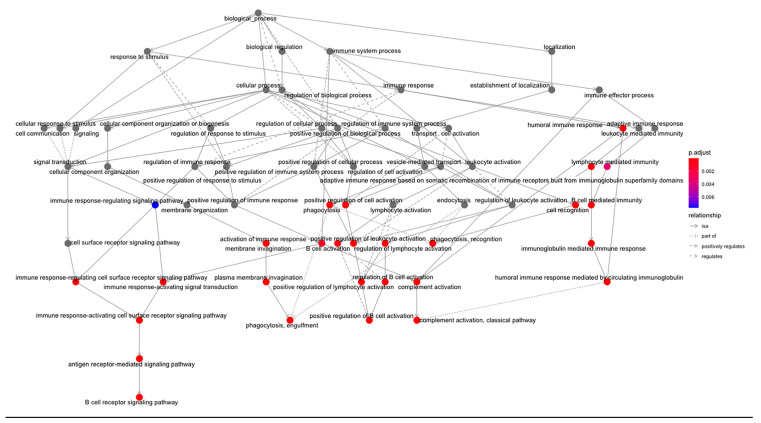
Gene ontology analysis from nodes with highest weights and bias in MLP constructed model. Analysis conducted using clusterProfiler—vector of genes returns the enriched ontologies at a *p*-value cutoff of 0.05 and q-value cutoff of 0.05. Pathway nodes are colored by significance.

To verify the clusters previously identified using SNF and CC, a Multi-Layer Perceptron (MLP) neural network was constructed, using the 1467 features selected by the univariate Cox regression model as node inputs. With three hidden layers, the output layer led to a classification into one of four clusters. This feedforward structure combined the selected feature inputs with initial weights and subjected them to an activation function for each layer (Figure 7A). The data were split into 80/20 randomized training/testing groups, and the model was evaluated on prediction accuracy. After model tuning, the final prediction accuracy was 91.3% (Figure 7B), with a confusion matrix demonstrating the test predictions (Figure 7C). Following model tuning and completion, the top nodes were isolated by filtering to those with the highest model weight and learning bias. Gene ontology analysis was then applied to those features, yielding many immune/inflammation-response-related pathways (Figure 8).

### 3.5. Signature Genes

**Figure 9:**
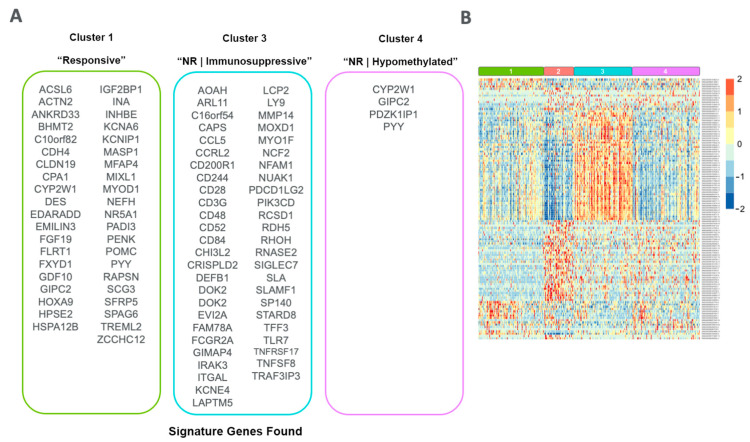
Signature gene sets found for each cluster. (**A**) List of overlapped DEGs and DMPs found from differential analysis comparing each cluster of interest. (**B**) Heatmap of the normalized (log2 FPKM + 1) expression counts for each signature gene, clustered by row and grouped by cluster.

Among the differentially expressed and methylated features of each cluster, a list of overlapping features occurring in both lists was produced (Figure 9A). The hierarchical clustering of these features, visualized by cluster, produced distinct patterns between each group (Figure 9B).

## 4. Discussion

Our findings have several notable implications, within the context of HGSOC genomic profiling. The semi-biased clustering of samples by integrated transcriptomic and methylomic data was superior to traditional hierarchical clustering, as well as feature selection using variability measures. The immunosuppressive phenotype found in Cluster 3 is distinct; the estimated presence of multiple stromal and immune cell types was associated with a poor response to treatment. Finally, the entire set of selected features was required for accurate subtype prediction using a supervised MLP neural network.

### 4.1. Clustering and Feature Selection

Since the wide deployment of next generation sequencing (NGS), several methods have been developed to cluster RNA sequencing data into de novo cancer subtypes. HGOSC, like other tumor types, is genetically and epigenetically heterogeneous [9]. The clustering of such data is further complicated by the presence of batch effects, inconsistent sequencing depth, differences in clinical features and treatment, data scarcity, and differences in assays. Thus, integrated clustering methods including multi-omic data types may offer insights which single-layer clustering cannot capture. RNA-seq data contain a larger number of total elements (typically ~60,000), and 27k methylation array data add an additional ~27,000 features; therefore, feature selection is critical toward developing a reproducible and effective model. Using all available features as input can negatively impact cluster accuracy and distinction [30]. Often, features are selected based on variability—using a measure of mean absolute deviation (MAD) across all samples [31,32,33]. Thus, the most variable features are used as inputs for unsupervised learning. However, the utility and interpretation of clusters resultant from such variable input features is unclear. Due to increased variability, the statistical power is also reduced. In our workflow, we forwent the MAD metric for clustering as we observed inferior separation between clusters tested (k = 2…8). Instead, a univariate Cox regression method was applied to select features most closely correlated with disease recurrence, across both layers of “omic” data. This resulted in 1467 total features selected for our analysis, with an almost equal representation of RNA-seq and methylomic features.

Multi-omics clustering methods used for subtyping have included basic k-means clustering, iCluster, similarity network fusion, and consensus clustering algorithms [33,34]. Such methods work well with high-dimensional data but are equally dependent upon the input data used for clustering. These methods largely assume a distribution of input feature data, e.g., Poisson (iCluster), negative binomial, and non-parametric (average-linkage hierarchical clustering). NMF clustering, or non-negative matrix factorization, utilizes dimensionality reduction to create a matrix as a proxy for clustering [34]. Intrinsically, NMF clustering can also only be used with non-negative values. Further, the utility of using these de novo clusters for clinical application beyond target discovery remains unclear.

Similarity network fusion (SNF) is a clustering approach which works best for multi-layer data integration [35]. This method works by building sample networks for each layer of data, then fusing them into one representative network. This approach has been used as the basis for more robust subtyping analyses in cancer [36,37]. Here, we demonstrate that pairing the SNF fusion matrix generated with aggregated consensus clustering (SNF + CC) results in more distinct clusters which are predictive of recurrence probability. Similar clustering methodology was used previously to define a novel subtype of sarcoma, which correlated with worse clinical outcomes [38].

We deem our approach “semi-biased” as the integrated features were selected based on their correlation with disease recurrence. Thus, the de novo clusters have an expected difference in predicted recurrence. However, by conducting downstream differential expression and methylation analysis between the most clinically different clusters, we identified additional features of each which may drive the phenotype. We also captured biological insights based on the differential analyses that were not evident with hierarchical clustering or a two-category definition of platinum response (i.e., “platinum-sensitive” vs. “platinum-resistant”). Finally, we performed a supervised neural network deep learning exercise to confirm the validity of our clusters and ensure the assignments were detectable based on the selected features alone. Each of the clinically distinct clusters (1—Responsive; 3—Non-Responsive, Immunosuppressive; 4—Non-Responsive, Hypomethylated) was associated with clinically significant findings.

### 4.2. Individual Findings Related to Each Defined Cluster

The Cluster 1 “Responsive” group contained multiple *SOX* genes which were downregulated (*SOX30*, *SOX 11*, *SOX3*). The sex-determining region Y-box (*SOX)* family of transcription factors has been linked to oncogenic processes, programs related to cell differentiation and stemness [39]. Some of the SOX genes have been associated with poor prognosis and cancer progression (*SOX2*, *SOX*5) [39,40]. In lung cancer, SOX30 had a tumor suppressor role through *p53* activation [41]. The high expression of SOX 30 was correlated with better overall survival. Similarly, *SOX11* has been implicated as a factor that can function as both a tumor suppressor and a metastasis inducer [42].

In contrast, *SOX3* was shown to upregulate pro-apoptotic genes in breast cancer and was associated with a poor prognosis. The SOX transcription factors can promote cell proliferation and survival in cancer in a context-dependent fashion. Of the three SOX genes found downregulated in Cluster 1, both *SOX11* and *SOX3* have been demonstrated to have oncogenic features in ovarian or cervical cancer [43,44,45]. We found all three genes downregulated in the cluster associated with the best clinical outcomes. Additionally, these genes were not differentially methylated in this cluster vs. the non-responsive clusters 3/4. This axis of transcription factors may function in a context-dependent fashion within this HGSOC subset.

The Cluster 3 “Non-Responsive|Immunosuppressive” group is noteworthy for its immune cell-type composition differences and its upregulation of multiple chemokine signaling and immunoglobulin heavy and kappa-chain-associated genes. Both *CXCR2* and *CCR2* have been associated with chemoresistance in multiple cancer types, including HGSOC [46]. The targeting of CXCR2 inhibited lung cancer progression and promoted the cytotoxic effects of cisplatin [47]. Many immune signaling chemokines and receptor genes were found (*CCL5*, *CCRL2*, *CD244*, *CD28*, *CD3G*, *CD48*, *CD52*, *CD84). IRAK3* has recently been studied for targeting to enhance pro-inflammatory cytokine signaling in cancer [48]. Immune cell-type composition analyses suggest interesting differences in stromal and immune cell composition in this cluster. Populations of macrophages, monocytic cells, and cancer-associated fibroblasts appeared to be increased in Cluster 3, as does the overall immune signature. Cancer-associated fibroblasts (CAFs) can promote tumor growth in the microenvironment via the remodeling of the extracellular matrix but also by the secretion of cytokines and growth factors, some of which were identified via differential analysis [49]. Macrophages can have both pro- and antitumoral effects depending on polarization state [50]. Tumor-associated macrophages (TAMs) can incline toward a pro-growth state, suppressing immune response by cytokine and chemokine production and the co-recruitment of other immunosuppressive cell types (e.g., myeloid-derived suppressor cells and regulatory T-cells) [51]. Interestingly, the activated myeloid dendritic cell (mDC) fraction was also higher in Cluster 3. Typically, mDCs function in antigen presentation and, when activated, can secrete IL-12 and promote the differentiation of T-cells into Th1 cells [52]. Thus, mDCs typically function in an immune-promoting fashion. Taken with the prior cell-type differences, the precise mechanism of immunosuppression in Cluster 3 warrants further examination.

The RNA-seq findings for Cluster 4 “Non-Responsive|Hypomethylated” were noteworthy in contrast to Cluster 3. The two groups had comparable clinical outcomes regarding both time to recurrence and time to death. Yet, whereas Cluster 3 showed the upregulation of multiple immunoglobulin chain coding genes, Cluster 4 showed the downregulation of this group. Both the mutation status of *IGHV* genes and expression response to treatment have been demonstrated as prognostic biomarkers [53]. Patients with a TP53 mutation and unmutated IGHV genes were predicted to have shorter time to first treatment in a cohort of chronic lymphocytic leukemia patients [54]. That the expression of these immunoglobulin genes should vary so much between two similar non-responsive groups was an unexpected finding, particularly as they were compared to the Cluster 1 (“Responsive”) baseline. Follow-up inspection is warranted around this gene set. Among the most upregulated DEGs in Cluster 4, which were not found in Cluster 3, we note *ELAVL3*, *GCK*, *PYY*, *SSTR3*, *FGF17*, and *PLPPR1*. Several of these have previously been noted for their varying roles in oncogenesis and cell proliferation [55,56,57,58]. *GCK* is a glucokinase responsible for glucose metabolism by sensing glucose uptake and encouraging insulin release. GCK is highly expressed in the liver, and its expression is closely related to hexokinase-2 [59]. Its role in ovarian cancer has not been studied. Our data support that the expression of this enzyme could be associated with worse clinical outcomes, potentially through the alteration of glucose metabolism.

The shared features of both non-responsive clusters (3 and 4) included the overexpression of multiple tumor antigen genes: *PAGE2*, *GAGE2A, GAGE13*, and *GAGE2E.* Such tumor antigens have been described in many cancer types, and they represent cancer/testis antigens which are normally expressed in male germ cells and are repressed in cancer [59,60]. They represent potential targets for innate or therapeutic immune recognition.

Notably, many DMPs within Cluster 4 were hypomethylated—compared to Cluster 1. This finding would imply a lack of epigenetic repression in the treatment-resistant group. We found that there were significantly more overexpressed genes in the DEG groups of both Cluster 4 and 3 (the *non-responsive* groups), compared to Cluster 1. Among the DMPs of Cluster 4, there were multiple anion transporters (*SLC16A3*, *SLC26A8*), as well as multiple altered probes in the promoter region of *IL1RN*. The latter gene codes for an interleukin 1 cytokine which modulates immune and inflammatory response [61].

Finally, our findings are restricted to HGSOC, a distinct entity within the spectrum of ovarian tumors, and confirm that the heterogeneity of clusters within HGSOC are unique to this disease. For instance, multiple studies have examined relapse propensity in low-grade serous ovarian cancer (LGSOC) [62,63]. LGSOC typically displays initial treatment resistance and a histology resembling more closely that of normally differentiated epithelial cells. Genomic profiling (*n* = 215) indicated high rates of *MAPK*, *BRAF*, and *NRAS* mutation. Notably, the overexpression of *CDKN2A* was associated with worse disease outcome, and the low expression of *CD274* (PDL1) was found in a subset of LGSOC patients. Conversely, other studies have demonstrated that high progesterone receptor expression, correlated with the presence of *CTNNB1* mutations, is associated with a favorable prognosis [64]. While low genomic complexity is a profile typical of LGSOC, our findings confirm the genetic and epigenetic complexity of HGSOC.

Further, the sheer number of features that were significantly correlated with recurrence in our Cox model supports the notion that a small group of genomic features are likely insufficient as a biomarker set for HGSOC prognostic classification. The clinical applicability of such models remains limited at this time; however, a validated signature including multiple genetic and epigenetic features, as we propose here, may become useful in the future to identify patients at high risk for early recurrence.

### 4.3. Utility and Data Limitations

The clinical utility of novel subtyping, even with the introduction of multi-omics integration and newer clustering methods, is still debated. Here, we present a novel workflow used to integrate methylation array data with NGS RNA-seq data. Using our semi-biased clustering approach, we were able to define gene signatures correlating with distinct phenotypes of chemo-response in subsets of HGSOC patients from TCGA and ICGC. We paired this approach with an MLP neural network learning model to ensure that each de novo cluster was predictable from the features selected for it. In so doing, we provide a feature selection, clustering, and deep learning approach which can be applied to any multi-omics dataset with a definable outcome measure (e.g., survival, recurrence, response to therapy). The inclusion of 27k array data presented challenging integration with RNA sequencing data. Firstly, the available probes are limited to the promoter region for this assay (distance to TSS ≤ 1500). Secondly, most genes are represented by only one probe—those with more probes had to be averaged by probe-wise methylation level. Finally, the normalization methods widely available for 450k and EPIC array are not available for 27k array data. Thus, a bespoke workflow had to be created for within-array normalization, probe-level quality filtration, and imputation.

## 5. Conclusions

Here, we utilize a workflow of integrated ‘omics data, across HGSOC tumors profiled in the TCGA to identify novel subtypes of the disease. Using unsupervised clustering, we present four unique phenotypes which are predictable via MLP machine learning architecture, based upon features most correlated with disease recurrence. Importantly, we identified genetic markers of each subtype, as well as a strong immunosuppressive signature in one cluster enriched for stromal and immune cell types. Our findings underscore the importance for ‘omic integration in single-cancer clustering analysis and can be extended to other layers of data.

## Figures and Tables

**Table 1:** SNF + CC clustering, subtype overview. The subtype classification, number of observations, and mean days to tumor recurrence are shown for each cluster (k = 4) identified. Three observations were removed due to missing data in at least one dataset.

Cluster	Observations (*n*)	Median Days to Tumor Recurrence	Median Days to Death	Subtype
1	101	977	1511	Responsive
2	50	723	1278	NM
3	98	568	1248	NR
4	111	428	1259	NR
*p*-Value: 6 × 10^−8^				

**Table 2:** Differentially expressed genes: Cluster 1 (“Responsive”) vs. All (A). Top 20 differentially expressed genes, sorted by significance (adjusted *p*-value). A total of 407 DEGs.

Gene	Log2 FC	Adj. *p*-Value	Type	Direction
GABRG2	−4.91896998	6.79 × 10^−27^	protein_coding	Downregulated
DPYSL5	−4.287431685	3.89 × 10^−26^	protein_coding	Downregulated
NXF2	−6.848164657	8.82 × 10^−26^	protein_coding	Downregulated
CUX2	−3.320974078	4.62 × 10^−21^	protein_coding	Downregulated
FGF17	−3.378578839	6.53 × 10^−21^	protein_coding	Downregulated
CLEC2L	−3.519088593	2.55 × 10^−19^	protein_coding	Downregulated
PAGE2	−5.530950007	2.55 × 10^−19^	protein_coding	Downregulated
IGF2BP1	−3.047958903	5.53 × 10^−18^	protein_coding	Downregulated
MAGEA9B	−5.083688527	2.53 × 10^−17^	protein_coding	Downregulated
IGDCC3	−3.087367559	2.64 × 10^−17^	protein_coding	Downregulated
OR3A2	−4.033030242	3.86 × 10^−17^	protein_coding	Downregulated
TNNT3	−2.729237962	1.31 × 10^−16^	protein_coding	Downregulated
ZNF716	−4.746030841	3.14 × 10^−16^	protein_coding	Downregulated
TUBB2B	−2.88250031	9.76 × 10^−16^	protein_coding	Downregulated
NXF2B	−5.151107593	1.23 × 10^−15^	protein_coding	Downregulated
IGLON5	−2.628183582	3.77 × 10^−15^	protein_coding	Downregulated
CYP2W1	−2.769837506	1.54 × 10^−14^	protein_coding	Downregulated
SLIT1	−2.276786555	4.87 × 10^−14^	protein_coding	Downregulated
RAPSN	−2.529386839	4.87 × 10^−14^	protein_coding	Downregulated
FAM133A	−2.672339049	7.34 × 10^−14^	protein_coding	Downregulated

**Table 3:** Differentially expressed genes: Cluster 3 (“Non-Responsive|Immunosuppressive”) vs. Cluster 1 (“Responsive”). Top 20 differentially expressed genes, sorted by significance (adjusted *p*-value). A total of 826 DEGs.

Gene	Log2 FC	Adj. *p*-Value	Type	Direction
IGHV3-72	4.85327615	1.01 × 10^−29^	IG_V_gene	Upregulated
IGLV7-46	4.10472706	3.87 × 10^−24^	IG_V_gene	Upregulated
IGHV4-34	3.87051574	9.83 × 10^−22^	IG_V_gene	Upregulated
IGKV1-39	4.89152857	1.19 × 10^−21^	IG_V_gene	Upregulated
IGLV1-47	3.86382442	9.44 × 10^−19^	IG_V_gene	Upregulated
IGKV1-12	3.95799068	8.33 × 10^−18^	IG_V_gene	Upregulated
IGHV1-58	5.2744758	8.33 × 10^−18^	IG_V_gene	Upregulated
IGHV4-28	4.0640693	5.68 × 10^−17^	IG_V_gene	Upregulated
PAGE2	6.19952601	8.28 × 10^−17^	protein_coding	Upregulated
IGHV2-5	3.70552433	1.24 × 10^−16^	IG_V_gene	Upregulated
IGHV1-18	3.64573859	4.17 × 10^−16^	IG_V_gene	Upregulated
IGHV1-2	3.85467877	6.57 × 10^−16^	IG_V_gene	Upregulated
IGHV3-49	3.89253991	1.76 × 10^−15^	IG_V_gene	Upregulated
IGHV3-20	4.7110679	3.41 × 10^−15^	IG_V_gene	Upregulated
IGLV9-49	4.0216455	5.63 × 10^−15^	IG_V_gene	Upregulated
IGHV5-51	3.47327279	1.22 × 10^−14^	IG_V_gene	Upregulated
P2RX1	2.08886429	2.98 × 10^−14^	protein_coding	Upregulated
IGKV5-2	4.30138825	3.27 × 10^−14^	IG_V_gene	Upregulated
IGHV3-73	4.1415814	3.55 × 10^−14^	IG_V_gene	Upregulated
IGHV3-11	3.51650267	5.26 × 10^−14^	IG_V_gene	Upregulated

**Table 4:** Differentially expressed genes: Cluster 4 (“Non-Responsive|Hypomethylated”) vs. Cluster 1 (“Responsive”). Top 20 differentially expressed genes, sorted by significance (adjusted *p*-value). A total of 225 DEGs.

Gene	Log2 FC	Adj. *p*-Value	Type	Direction
*GAGE2E*	5.63658378	6.82 × 10^−6^	protein_coding	Upregulated
*CTAG1A*	5.24453883	9.92 × 10^−6^	protein_coding	Upregulated
*CTAG1B*	5.15421251	1.84 × 10^−9^	protein_coding	Upregulated
*NXF2*	5.04533375	6.06 × 10^−8^	protein_coding	Upregulated
*PAGE2*	4.95316397	4.13 × 10^−10^	protein_coding	Upregulated
*GAGE13*	4.92955607	1.53 × 10^−5^	protein_coding	Upregulated
*NXF2B*	4.78873403	8.63 × 10^−9^	protein_coding	Upregulated
*GAGE2A*	4.4743878	0.00088715	protein_coding	Upregulated
*GABRG2*	4.3361448	1.54 × 10^−13^	protein_coding	Upregulated
	3.93401196	1.36 × 10^−5^	lncRNA	Upregulated
*SPRR1B*	−3.4839398	0.00023679	protein_coding	Downregulated
*IGHV2-26*	−3.1670253	3.89 × 10^−8^	IG_V_gene	Downregulated
*IGLV3-1*	−3.1480808	1.55 × 10^−9^	IG_V_gene	Downregulated
*IGLV3-21*	−3.1374207	3.76 × 10^−10^	IG_V_gene	Downregulated
*IGHV5-10-1*	−2.9508194	4.33 × 10^−5^	IG_V_gene	Downregulated
*IGLV3-27*	−2.9179351	9.52 × 10^−8^	IG_V_gene	Downregulated
*IGHV3-64D*	−2.6754653	0.00046012	IG_V_gene	Downregulated
*IGLV2-23*	−2.5860868	9.12 × 10^−7^	IG_V_gene	Downregulated
*IGHV3-7*	−2.5576913	1.29 × 10^−6^	IG_V_gene	Downregulated
*IGHV3-13*	−2.4825443	0.00010162	IG_V_gene	Downregulated

## Data Availability

TCGA expression and methylation data are available through GDC portal: https://portal.gdc.cancer.gov.

## Supplementary Materials

The following supporting information can be downloaded at: https://www.mdpi.com/article/10.3390/cancers15143649/s1, Figure S1. Results of Consensus Clustering of RNA and 450k array features from ICGC validation dataset, selected by Cox Regression (*n* = 6884 gene features). KM-based recurrence-risk analysis performed for each identified cluster. Recurrence variable analyzed against the censorship variable (lost to follow-up, study withdrawal). K = 4 clusters were optimal for significance (*p*-value < 0.0001). Table S1. Top 20 features selected by Cox regression model (*p* < 0.05) of integrated transcriptomic and methylomic TCGA data. Table S2. Results of Kruskal-Wallis test, comparing the mean of various immune cell-type fractions across all identified clusters. Highlighted rows have a *p*-value < 0.01. Table S3. Common features selected in both primary analysis of integrated TCGA data, and validation analysis of integrated ICGC data. ICGC 450k methylation array data were limited to promoter-associated features (TSS +/− 1500) when examining overlapped features.

## References

[1] Ovary Statistics|American Cancer Society—Cancer Facts & Statistics. https://cancerstatisticscenter.cancer.org/#!/cancer-site/Ovary.

[2] (2023). SEER Ovarian Cancer. https://seer.cancer.gov/statfacts/html/ovary.html.

[3] Lisio M.-A., Fu L., Goyeneche A., Gao Z.-H., Telleria C. (2019). High-Grade Serous Ovarian Cancer: Basic Sciences, Clinical and Therapeutic Standpoints. Int. J. Mol. Sci..

[4] Nero C., Vizzielli G., Lorusso D., Cesari E., Daniele G., Loverro M., Scambia G., Sette C. (2021). Patient-derived organoids and high grade serous ovarian cancer: From disease modeling to personalized medicine. J. Exp. Clin. Cancer Res..

[5] Matulonis U.A., Shapira-Frommer R., Santin A.D., Lisyanskaya A.S., Pignata S., Vergote I., Raspagliesi F., Sonke G.S., Birrer M., Provencher D.M. (2019). Antitumor activity and safety of pembrolizumab in patients with advanced recurrent ovarian cancer: Results from the phase II KEYNOTE-100 study. Ann. Oncol..

[6] Fleming G.F., Brunetto V.L., Cella D., Look K.Y., Reid G.C., Munkarah A.R., Kline R., Burger R.A., Goodman A., Burks R.T. (2004). Phase III Trial of Doxorubicin Plus Cisplatin with or without Paclitaxel Plus Filgrastim in Advanced Endometrial Carcinoma: A Gynecologic Oncology Group Study. J. Clin. Oncol..

[7] Mannel R.S., Brady M.F., Kohn E.C., Hanjani P., Hiura M., Lee R., DeGeest K., Cohn D.E., Monk B.J., Michael H. (2011). A randomized phase III trial of IV carboplatin and paclitaxel × 3 courses followed by observation versus weekly maintenance low-dose paclitaxel in patients with early-stage ovarian carcinoma: A Gynecologic Oncology Group Study. Gynecol. Oncol..

[8] Berg T., Nøttrup T.J., Roed H. (2019). Gemcitabine for recurrent ovarian cancer—A systematic review and meta-analysis—PubMed. Gynecol. Oncol..

[9] The Cancer Genome Atlas Research Network (2011). Integrated genomic analyses of ovarian carcinoma. Nature.

[10] Tothill R.W., Tinker A.V., George J., Brown R., Fox S.B., Lade S. (2008). Novel molecular subtypes of serous and endometrioid ovarian cancer linked to clinical outcome. Clin. Cancer Res..

[11] Konecny G.E., Wang C., Hamidi H., Winterhoff B., Kalli K.R., Dering J., Ginther C., Chen H.W., Dowdy S., Cliby W. (2014). Prognostic and therapeutic relevance of molecular subtypes in high-grade serous ovarian cancer. J. Natl. Cancer Inst..

[12] McCluggage W.G. (2011). Morphological subtypes of ovarian carcinoma: A review with emphasis on new developments and pathogenesis. Pathology.

[13] Barnes B.M., Nelson L., Tighe A., Burghel G.J., Lin I.-H., Desai S., McGrail J.C., Morgan R.D., Taylor S.S. (2021). Distinct transcriptional programs stratify ovarian cancer cell lines into the five major histological subtypes. Genome Med..

[14] Riso P.L., Villa C.E., Gasparoni G., Vingiani A., Luongo R., Manfredi A., Jungmann A., Bertolotti A., Borgo F., Garbi A. (2020). A cell-of-origin epigenetic tracer reveals clinically distinct subtypes of high-grade serous ovarian cancer. Genome Med..

[15] Grossman R.L., Heath A.P., Ferretti V. (2016). Genomic Data Commons: A Data-Sharing Platform and Analytic Toolset for Consensus-Oriented Research. Am. J. Hum. Genet..

[16] Li B., Dewey C.N. (2011). RSEM: Accurate transcript quantification from RNA-Seq data with or without a reference genome. BMC Bioinform..

[17] Tian Y., Morris T.J., Webster A.P., Yang Z., Beck S., Feber A., Teschendorff A.E. (2017). ChAMP: Updated methylation analysis pipeline for Illumina BeadChips. Bioinformatics.

[18] Deo S.V., Deo V., Sundaram V. (2021). Survival analysis—part 2: Cox proportional hazards model. Indian J. Thorac. Cardiovasc. Surg..

[19] Therneau T.M., Grambsch P.M. (2000). Modeling Survival Data: Extending the Cox Model.

[20] Xu D., Tian Y. (2015). A Comprehensive Survey of Clustering Algorithms. Ann. Data Sci..

[21] Xu T., Le T.D., Liu L., Su N., Wang R., Sun B., Colaprico A., Bontempi G., Li J. (2017). CancerSubtypes: An R/Bioconductor package for molecular cancer subtype identification, validation and visualization. Bioinformatics.

[22] Love M.I., Huber W., Anders S. (2014). Moderated estimation of fold change and dispersion for RNA-seq data with DESeq2. Genome Biol..

[23] Ritchie M.E., Phipson B., Wu D., Hu Y., Law C.W., Shi W., Smyth G.K. (2015). limma powers differential expression analyses for RNA-sequencing and microarray studies. Nucleic Acids Res..

[24] Kuleshov M.V., Jones M.R., Rouillard A.D., Fernandez N.F., Duan Q., Wang Z., Koplev S., Jenkins S.L., Jagodnik K.M., Lachmann A. (2016). Enrichr: A comprehensive gene set enrichment analysis web server 2016 update. Nucleic Acids Res..

[25] Hung J., Goodman A., Ravel D., Lopes S.C.P., Rangel G.W., Nery O.A., Malleret B., Nosten F., Lacerda M.V.G., Ferreira M.U. (2020). Keras R-CNN: Library for cell detection in biological images using deep neural networks. BMC Bioinform..

[26] Wu T., Hu E., Xu S., Chen M., Guo P., Dai Z., Feng T., Zhou L., Tang W., Zhan L. (2021). clusterProfiler 4.0: A universal enrichment tool for interpreting omics data. Innovation.

[27] Cedoz P.-L., Prunello M., Brennan K., Gevaert O. (2018). MethylMix 2.0: An R package for identifying DNA methylation genes. Bioinformatics.

[28] Sturm G., Finotello F., List M. (2020). Immunedeconv: An R Package for Unified Access to Computational Methods for Estimating Immune Cell Fractions from Bulk RNA-Sequencing Data. Bioinform. Cancer Immunother..

[29] Hudson T.J., Anderson W., Artez A., Barker A.D., Bell C., Bernabé R.R., Bhan M.K., Calvo F., Eerola I., Gerhard D.S. (2010). International Network of Cancer Genome Projects. Nature.

[30] Tritchler D., Parkhomenko E., Beyene J. (2009). Filtering Genes for Cluster and Network Analysis. BMC Bioinform..

[31] Townes F.W., Hicks S.C., Aryee M.J., Irizarry R.A. (2019). Feature selection and dimension reduction for single-cell RNA-Seq based on a multinomial model. Genome Biol..

[32] Wenric S., Shemirani R. (2018). Using Supervised Learning Methods for Gene Selection in RNA-Seq Case-Control Studies. Front. Genet..

[33] Lim D.K., Rashid N.U., Ibrahim J.G. (2021). Model-based feature selection and clustering of RNA-seq data for unsupervised subtype discovery. Ann. Appl. Stat..

[34] Gaujoux R., Seoighe C. (2010). A flexible R package for nonnegative matrix factorization. BMC Bioinform..

[35] Wang B., Mezlini A.M., Demir F., Fiume M., Tu Z., Brudno M., Haibe-Kains B., Goldenberg A. (2014). Similarity network fusion for aggregating data types on a genomic scale. Nat. Methods.

[36] Chiu A.M., Mitra M., Boymoushakian L., Coller H.A. (2018). Integrative analysis of the inter-tumoral heterogeneity of triple-negative breast cancer. Sci. Rep..

[37] Ramazzotti D., Lal A., Wang B., Batzoglou S., Sidow A. (2018). Multi-omic tumor data reveal diversity of molecular mechanisms that correlate with survival. Nat. Commun..

[38] Zhu Z., Jin Z., Zhang H., Zhang M., Sun D. (2020). Integrative Clustering Reveals a Novel Subtype of Soft Tissue Sarcoma with Poor Prognosis. Front. Genet..

[39] Grimm D., Bauer J., Wise P., Krüger M., Simonsen U., Wehland M., Infanger M., Corydon T.J. (2019). The role of SOX family members in solid tumours and metastasis. Semin. Cancer Biol..

[40] Zou H., Wang S., Wang S., Wu H., Yu J., Chen Q., Cui W., Yuan Y., Wen X., He J. (2018). SOX5 interacts with YAP1 to drive malignant potential of non-small cell lung cancer cells. Am. J. Cancer Res..

[41] Han F., Liu W., Jiang X., Shi X., Yin L., Ao L., Cui Z., Li Y., Huang C., Cao J. (2014). SOX30, a novel epigenetic silenced tumor suppressor, promotes tumor cell apoptosis by transcriptional activating p53 in lung cancer. Oncogene.

[42] Yang Z., Jiang S., Lu C., Ji T., Yang W., Li T., Lv J., Hu W., Yang Y., Jin Z. (2019). SOX11: Friend or foe in tumor prevention and carcinogenesis?. Ther. Adv. Med. Oncol..

[43] Li X., Wu X., Li Y., Cui Y., Tian R., Singh N., Ding M., Yang Y., Gao Y. (2019). Promoter hypermethylation of SOX11 promotes the progression of cervical cancer in vitro and in vivo. Oncol. Rep..

[44] Paskeh M.D.A., Mirzaei S., Gholami M.H., Zarrabi A., Zabolian A., Hashemi M., Hushmandi K., Ashrafizadeh M., Aref A.R., Samarghandian S. (2021). Cervical cancer progression is regulated by SOX transcription factors: Revealing signaling networks and therapeutic strategies. Biomed. Pharmacother..

[45] Farris C., Zsiros E., Lynam S., Kelley J.L., Bristow R.E. (2019). Interrogation of transcriptional factors in ovarian cancer subtypes identifies Sox11 as a potential therapeutic target. Cancer Epidemiol. Biomarkers Prev..

[46] Liang Z., Zhan W., Zhu A., Yoon Y., Lin S., Sasaki M. (2012). CXCR2 promotes ovarian cancer growth through dysregulated cell cycle, diminished apoptosis, and enhanced angiogenesis. Clin. Cancer Res..

[47] Cheng Y., Mo F., Li Q., Han X., Shi H., Chen S., Wei Y., Wei X. (2021). Targeting CXCR2 inhibits the progression of lung cancer and promotes therapeutic effect of cisplatin. Mol. Cancer..

[48] Freihat L.A., Wheeler J.I., Wong A., Turek I., Manallack D.T., Irving H.R. (2019). IRAK3 modulates downstream innate immune signalling through its guanylate cyclase activity. Sci. Rep..

[49] Kalluri R. (2016). The biology and function of fibroblasts in cancer. Nat. Rev. Cancer.

[50] Mantovani A., Allavena P. (2015). The interaction of anticancer therapies with tumor-associated macrophages. J. Exp. Med..

[51] Noy R., Pollard J.W. (2014). Tumor-associated macrophages: From mechanisms to therapy. Immunity.

[52] Banchereau J., Steinman R.M. (1998). Dendritic cells and the control of immunity. Nature.

[53] Li X., Wu N., Li B. (2019). A high mutation rate of immunoglobulin heavy chain variable region gene associates with a poor survival and chemotherapy response of mantle cell lymphoma patients. Medicine.

[54] Delgado J., Salaverria I., Baumann T., Martínez-Trillos A., Lee E., Jiménez L., Navarro A., Royo C., Santacruz R., López C. (2014). Genomic complexity and IGHV mutational status are key predictors of outcome of chronic lymphocytic leukemia patients with TP53 disruption. Haematologica.

[55] Těšínský M., Šimčíková D., Heneberg P. (2019). First evidence of changes in enzyme kinetics and stability of glucokinase affected by somatic cancer-associated variations. Biochim. Biophys. Acta Proteins Proteom..

[56] Heer R., Douglas D., Mathers M., Robson C., Leung H. (2004). Fibroblast growth factor 17 is over-expressed in human prostate cancer. J. Pathol..

[57] Lee M., Lupp A., Mendoza N., Martin N., Beschorner R., Honegger J., Schlegel J., Shively T., Pulz E., Schulz S. (2014). SSTR3 is a putative target for the medical treatment of gonadotroph adenomas of the pituitary. Endocr. Relat. Cancer.

[58] Tseng W.W., Liu C.D. (2002). Peptide YY and cancer: Current findings and potential clinical applications. Peptides.

[59] DeWaal D., Nogueira V., Terry A.R., Patra K.C., Jeon S.M., Guzman G., Au J., Long C.P., Antoniewicz M.R., Hay N. (2018). Hexokinase-2 depletion inhibits glycolysis and induces oxidative phosphorylation in hepatocellular carcinoma and sensitizes to metformin. Nat. Commun..

[60] Mahmoud A.M. (2018). Cancer testis antigens as immunogenic and oncogenic targets in breast cancer. Immunotherapy.

[61] Janicki L., Schierwagen R., Sauerbruch T., Wree A. (2017). Interleukin-1 receptor antagonist modulates the early phase of liver regeneration after partial hepatectomy in mice. PLoS ONE.

[62] Gershenson D.M., Cobb L.P., Westin S.N., Zhang Y., Jazaeri A., Malpica A., Sun C.C. (2022). Contemporary primary treatment of women with stage II–IV low-grade serous ovarian/peritoneal cancer (LGSOC): Determinants of relapse and disease-free survival. Gynecol. Oncol..

[63] Gershenson D.M., Sun C.C., Westin S.N., Eyada M., Cobb L.P., Nathan L.C., Sood A.K., Malpica A., Hillman R.T., Wong K.K. (2022). The genomic landscape of low-grade serous ovarian/peritoneal carcinoma and its impact on clinical outcomes. Gynecol. Oncol..

[64] Hollis R.L. (2023). Molecular characteristics and clinical behaviour of epithelial ovarian cancers. Cancer Lett..

