# Supplementary material for: Integrated Multi-Omic Analysis Reveals Immunosuppressive Phenotype Associated with Poor Outcomes in High-Grade Serous Ovarian Cancer
Source: Cancers (Basel). 2023 Jul 17;15(14):3649. doi: 10.3390/cancers15143649 (PMC10377286; doi:10.3390/cancers15143649)
Supplement: Supplementary file 1 [file cancers-15-03649-s001.zip › cancers-2430574-supplementary.pdf]

**Supplemental Table S1.**

| **Feature ID** | **Gene** | **p-value** |
|---|---|---|
| cg21022435 | NFRKB | 8.88E-06 |
| cg10835876 | DPT | 3.33E-05 |
| ENSG00000161911 | TREML1 | 1.31E-04 |
| cg13406768 | C2orf33 | 2.37E-04 |
| ENSG00000228058 | LINC01736 | 2.40E-04 |
| cg08278554 | C15orf48 | 2.44E-04 |
| ENSG00000238110 | | 2.72E-04 |
| cg06351503 | RDBP | 2.79E-04 |
| cg25274750 | HIPK2 | 3.63E-04 |
| ENSG00000287012 | | 3.76E-04 |
| ENSG00000235437 | LINC01278 | 3.87E-04 |
| ENSG00000115592 | PRKAG3 | 4.15E-04 |
| ENSG00000256849 | TCP1P3 | 4.16E-04 |
| ENSG00000288302 | | 4.39E-04 |
| cg14802310 | TUBA3 | 4.48E-04 |
| cg08377000 | TIGD2 | 4.82E-04 |
| ENSG00000287680 | | 4.86E-04 |
| ENSG00000105131 | EPHX3 | 6.16E-04 |
| ENSG00000287255 | | 6.42E-04 |
| cg20655558 | DNAJB7 | 6.99E-04 |

***Supplemental Table S1.*** *Top 20 features selected by Cox regression model ($p < 0.05$) of integrated transcriptomic and methylomic TCGA data.*

## Supplemental Figure S1.

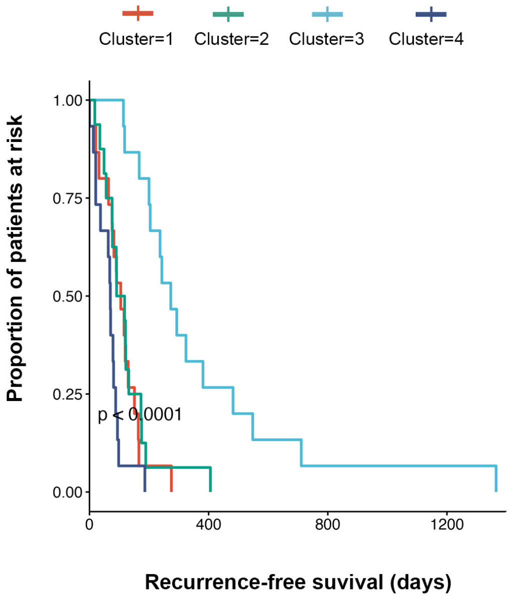


***Supplemental Figure S1.*** *Results of Consensus Clustering of RNA and 450k array features from ICGC validation dataset, selected by Cox Regression (n = 6,884 gene features). KM-based recurrence-risk analysis performed for each identified cluster. Recurrence variable analyzed against the censorship variable (lost to follow-up, study withdrawal). K = 4 clusters were optimal for significance (p-value < 0.0001).*

## Supplemental Table S2.

| Cell Type | Kruskal-Wallis P-Val |
|---|---|
| Myeloid dendritic cell activated | 2.20E-16 |
| B cell | 1.20E-10 |
| T cell CD4+ memory | 3.80E-05 |
| T cell CD4+ naive | 1.50E-04 |
| T cell CD4+ (non-regulatory) | 6.40E-01 |
| T cell CD4+ central memory | 3.90E-01 |
| T cell CD4+ effector memory | 2.20E-16 |
| T cell CD8+ naive | 1.00E-02 |
| T cell CD8+ | 1.80E-11 |
| T cell CD8+ central memory | 2.20E-16 |
| T cell CD8+ effector memory | 1.90E-04 |
| Class-switched memory B cell | 6.50E-11 |
| Common lymphoid progenitor | 8.60E-02 |
| Common myeloid progenitor | 7.20E-01 |
| Myeloid dendritic cell | 2.20E-16 |
| Endothelial cell | 8.50E-03 |
| Eosinophil | 6.20E-02 |
| Cancer associated fibroblast | 2.60E-03 |
| Granulocyte-monocyte progenitor | 1.10E-01 |
| Hematopoietic stem cell | 1.80E-03 |
| Macrophage | 2.20E-16 |
| Macrophage M1 | 2.20E-16 |
| Macrophage M2 | 2.20E-16 |
| Mast cell | 8.00E-05 |
| B cell memory | 4.00E-02 |
| Monocyte | 2.20E-16 |
| B cell naive | 6.00E-01 |
| Neutrophil | 6.00E-01 |
| NK cell | 5.20E-01 |
| T cell NK | 9.50E-05 |
| Plasmacytoid dendritic cell | 2.20E-16 |
| B cell plasma | 4.40E-02 |
| T cell gamma delta | 1.10E-01 |
| T cell CD4+ Th1 | 2.30E-05 |
| T cell CD4+ Th2 | 3.20E-01 |
| T cell regulatory (Tregs) | 5.10E-01 |
| immune score | 2.20E-16 |
| stroma score | 2.00E-04 |
| microenvironment score | 2.20E-16 |

***Supplemental Table S2.*** *Results of Kruskal-Wallis test, comparing the mean of various immune cell-type fractions across all identified clusters. Highlighted rows have a p-value < 0.01.*

## Supplemental Table S3.

| Alias | Gene | Description |
|---|---|---|
| ENSG00000060762 | MPC1 | mitochondrial pyruvate carrier 1 |
| ENSG00000100075 | SLC25A1 | solute carrier family 25 member 1 |
| ENSG00000100263 | RHBDD3 | rhomboid domain containing 3 |
| ENSG00000123472 | ATPAF1 | ATP synthase mitochondrial F1 complex assembly factor 1 |
| ENSG00000138286 | FAM149B1 | family with sequence similarity 149 member B1 |
| ENSG00000141622 | RNF165 | ring finger protein 165 |
| ENSG00000144230 | GPR17 | G protein-coupled receptor 17 |
| ENSG00000149798 | CDC42EP2 | CDC42 effector protein 2 |
| ENSG00000169093 | ASMTL | acetylserotonin O-methyltransferase like |
| | | |
| **Probe ID** | **Gene** | **CGI Position** |
| cg00265812 | UXT | Island |
| cg00702231 | HYMAI | Island |
| cg00720747 | GLIS2 | N_Shore |
| cg01151424 | KCNJ11 | Island |
| cg01741626 | GLIS2 | Island |
| cg01784327 | SPON2 | S_Shore |
| cg01970906 | CUL4A | Island |
| cg02476461 | SEMA5A | N_Shore |
| cg03124680 | NAP1L4 | Island |
| cg03238901 | OLFM2 | Island |
| cg03322234 | CYP2W1 | N_Shore |
| cg04423342 | SEMA4G | N_Shore |
| cg04840930 | HYMAI | S_Shore |
| cg05886087 | IL1R1 | N_Shore |
| cg06206471 | GLIS2 | Island |
| cg06550951 | HIPK2 | Island |
| cg07077459 | HYMAI | Island |
| cg07659663 | CHID1 | S_Shelf |
| cg07865580 | UXT | Island |
| cg08134678 | ARHGAP9 | S_Shelf |
| cg08263357 | HYMAI | Island |
| cg08327532 | CHAT | Island |
| cg08841829 | ABCG1 | N_Shore |
| cg10007452 | HYMAI | Island |
| cg11287660 | C15orf48 | Island |
| cg11306587 | NDRG4 | Island |
| cg12072001 | SPON2 | S_Shore |
| cg12271199 | JUN | Island |

| | | |
|---|---|---|
| cg12757684 | HYMAI | Island |
| cg13151664 | KALRN | NA |
| cg14582226 | CAV1 | N_Shore |
| cg14736837 | ASB11 | NA |
| cg14908186 | CUL4A | Island |
| cg15914863 | CYP2W1 | N_Shore |
| cg16093752 | RAC3 | Island |
| cg16785050 | NAP1L4 | Island |
| cg18349138 | CHODL | Island |
| cg18642179 | MGMT | N_Shore |
| cg19606431 | ENPP3 | Island |
| cg20088969 | DDIT4 | N_Shore |
| cg20811266 | CAV1 | N_Shore |
| cg21135533 | PRDM14 | Island |
| cg21522636 | RASSF1 | Island |
| cg21545785 | UXT | Island |
| cg22352234 | HYMAI | Island |
| cg22378065 | HYMAI | Island |
| cg22573917 | WNT1 | N_Shore |
| cg23460430 | HYMAI | S_Shore |
| cg23635374 | SERPINH1 | Island |
| cg24885417 | NPY | Island |
| cg25350411 | HYMAI | Island |
| cg25635916 | SEMA3B | N_Shelf |
| cg25719876 | TSPAN18 | NA |
| cg26090107 | SLC15A3 | Island |
| cg27002185 | CDKN1C | Island |
| cg27370461 | PPP2R1B | Island |

***Supplemental Table S3.*** *Common features selected in both primary analysis of integrated TCGA data, and validation analysis of integrated ICGC data. ICGC 450k methylation array data were limited to promoter-associated features (TSS +/- 1500) when examining overlapped features.*
